# Editorial: The role, and underlying neural mechanisms of, physical activity in treating substance and alcohol use disorders

**DOI:** 10.3389/fnint.2025.1589088

**Published:** 2025-05-30

**Authors:** Kolter B. Grigsby, Zaynah S. Usmani, Christina J. Perry, Angela R. Ozburn

**Affiliations:** ^1^Department of Pharmaceutical Sciences, Washington State University, Spokane, WA, United States; ^2^Portland Veterans Affairs Medical Center, Research and Development Service, Portland, OR, United States; ^3^Department of Behavioral Neuroscience, Oregon Health and Science University, Portland, OR, United States; ^4^The Florey Institute of Neuroscience and Mental Health, Parkville, VIC, Australia; ^5^Florey Department of Neuroscience and Mental Health, Parkville, VIC, Australia

**Keywords:** adjunctive therapeutic treatment, physical activity, high fat feeding (HFF), alcohol use disorder (AUD), acupuncture

## Introduction

Rodent models have long been instrumental in uncovering the complex physiological and psychological processes driving human disease. However, our understanding of non-invasive and non-pharmacological interventions—those long deemed “alternative”—are sorely lacking. Voluntary wheel-running behavior in rodents serves as a valuable lens through which to explore the critical role of physical activity (PA) as a cornerstone of human health. Moreover, there is growing interest and clear need to investigate the mechanisms underlying traditional treatment options, like acupuncture. The complimentary contributions of this special topic investigate the interplay between everyday health factors—namely stress, PA levels, high-fat diet, and alcohol use—and the unique opportunity to implement adjunctive, non-invasive interventions to improve health.

## Investigating neural and behavioral barriers to PA

Growing evidence suggests that adverse life experiences can negatively impact long-term PA levels (Moraska and Fleshner, 2001; Stults-Kolehmainen and Sinha, 2014; DeVallance et al., 2017; Chauntry et al., 2022; Raney et al., 2022). Yet, the biological factors mediating the connection between stress and PA motivation remain unclear. The contributed work of Buhr et al. presents several novel findings that may help elucidate this complex relationship. Here, wheel running was found to be inversely proportional to the number of tail shocks received, indicating a negative relationship between stress intensity and PA engagement. Similarly, stress exposure reduced the latency to reach failure on a graded treadmill test—a reliable measure of reduced PA performance, which may reflect features of low PA motivation. Follow-up testing found that tail-shock blunted PA-induced reductions in dopamine (DA) and serotonin (5HT) processing that were consistent across several brain regions. These findings reflect known adaptive stress-coping mechanisms in response to PA, which warrant further testing as potential neurobiological barriers to PA engagement (Dishman et al., 1997; Greenwood and Fleshner, 2011; Mul et al., 2018). Burh et al. also found that stress exposure increased HSP70 and decreased SOD2 protein concentrations in skeletal muscle—markers of prolonged oxidative stress and inflammation—that were otherwise unaltered by PA. Taken together, Buhr et al. provide critical insights into behavioral, central, and peripheral responses to adverse experiences that may mediate long-term PA deficits.

Despite known benefits of regular exercise, physical inactivity remains a global issue (Ruegsegger and Booth, 2018; Katzmarzyk, 2023). To address the insufficient biological understanding of exercise adherence, it may be important to consider the physiological stress of PA itself as a potential motivational barrier. Forced models of PA rodents, such as treadmill running, are largely considered more physiologically stressful than voluntary models (i.e., wheel-running). However, consistence evidence suggests that acute WR (1–2 weeks) evokes physiological and neurobiological markers of stress, whereas more chronic stages of voluntary PA (4-weeks) do not (Fediuc et al., 2006; Grigsby et al., 2022). In their contribution, Grigsby et al. introduced the 3D-printable, “Dependable, Simple, and Cost-effective (DSC)” running wheel as an affordable and precise means of exploring distinct running characteristics at key stages of PA reinforcement (Grigsby et al.); wherein wheel-running patterns at acute and chronic timepoints were characterized in a mouse model of harmful ethanol drinking (inbred High Drinking in the Dark; iHDID-1) and their heterogenous founders (Heterogenous Stock/Northport; HS/Npt). For both genotypes, there was a consistent increase in the average running distance from day 1 to day 14 of wheel access, which was maintained until day 28. This mirrors foundational wheel studies in mice and rats, which similarly show a shift toward habitual running patterns over time. In support of prior work (McCulley et al., 2013), iHDID-1 displayed less fluctuation in their 24-h running pattern compared to HS/Npt mice. Additional running characteristics, such as daily running distance, running time, and circadian actograms, were otherwise the same between both genotypes (and across both sexes; Grigsby et al.). In all, Grigsby et al. showcase the DSC wheel as a customizable, precise, and cost-effective tool for exploring PA behavior in real-time.

## Sex-specific differences in PA and diet

Human disease is complex and often undifferentiating—studying both sexes is a clear and basic first step toward finding inclusive and effective medical treatments. Sex differences in rodent and human PA depend on genetic background, age, and environmental influences. Female rodents tend to run greater distances and at higher intensities than males; however, some mouse strains (i.e., CBA/J and CAST/Ei) show comparable running patterns between sexes (Lightfoot et al., 2004; Ghosh et al., 2010). The present contribution from Grigsby et al. also found little sex differences in wheel activity in iHDID-1 and HS/Npt mice. Despite their well-known strain difference in binge-like ethanol intake, few sex-specific differences in ethanol-related measures across iHDID-1 and HS/Npt mice have been noted (Crabbe et al., 2014; Jensen et al., 2021; Savarese et al., 2021). Expanding our understanding of the role of sex in PA and other patterned behaviors, such as substance use and high-fat feeding, is crucial for effectively guiding PA as an adjunctive therapy in the future.

The work provided by Kocum et al. investigated the effects of voluntary PA on diet preference between a palatable high-fat diet and a less palatable, nutritionally balanced diet in male and female Sprague-Dawley rats (Kocum et al.). Wheel access differentially shifted diet preference and consumption based on sex, wherein sedentary females consumed 20% less high-fat diet compared to their PA counterparts—indicative of an adaptive response to sedentary behavior. In contrast, males displayed a maladaptive, 50% increase in high-fat diet consumption under sedentary conditions. In both sexes, the effects of PA on diet preference were reversible within 24 h of alternating wheel conditions. Intriguingly, no correlation was found between running distance and diet intake, suggesting wheel access alone (not PA, *per se*) may influence feeding behavior. Kocum et al. further described sex-specific differences in striatal opioid and dopamine-related genes in response to PA and diet, with males showing increased expression of *Drd2* and *Penk* and females showing decreased expression of *Drd2, Opmr1*, and *Penk*. These largely opposite gene responses may in-turn underscore sex-differences in sensitivity to the reinforcing properties of palatable food and PA. Taken together, this critical work underscores the complicated interplay between sex, sedentary behavior, and overconsumption of palatable foods.

## Exploring adjunctive therapies in the context of alcohol use disorder

There are only three pharmacotherapies for U.S. citizens struggling with Alcohol Use Disorder (AUD). For some, these options are ineffective, cost prohibitive, or harbor side effects. Finding adjunctive, non-invasive treatment options that can complement existing therapies will undoubtedly act to offset these major downsides.

Voluntary ethanol intake and voluntary wheel-running have been widely studied since the early 20th century (Stewart, 1898; Hausmann, 1932). Furthermore, the relationship between PA and harmful drinking has been explored across species, strains, and in both sexes (Ozburn et al., 2008; Ehringer et al., 2009; Booher et al., 2019; Buhr et al., 2021). Although not directly addressed by Grigsby et al., future exploration of how these behaviors interact (in real-time) in iHDID mice could help better understand the potential of PA to regulate harmful drinking.

Acupuncture—which has been practiced in China since 2500 BC—has been explored as a potential treatment option for substance use disorders for decades (McLellan et al., 1993; Tan et al., 2025). For instance, implementation of a protocol developed by the US National Acupuncture Detoxification Association (NADA) has been shown to be effective in treating cocaine and opiate misuse in humans (Bullock et al., 1999; Lin et al., 2012). Acupuncture has similarly emerged as a potential intervention for alcohol dependence and related symptomology (Zhao et al., 2014; Chang et al., 2019). The contributed work of Seo et al. explored the effects of stimulating the HT7 (Shenmen) acupuncture point (in an anatomically analogous location) on ethanol intake in rats. Moreover, Seo et al. further examined microglia-related responses across the medial prefrontal cortex (mPFC), habenula (Hb), and ventral tegmental area (VTA) in response to Shenmen-like acupuncture. The results found that HT7 acupuncture significantly reduced ethanol consumption. Seo et al. further observed that HT7 stimulation blunted ethanol-induced increases in protein markers of microglia activity (Arginase-1, Iba-1, and Sigma-1) within the aforementioned brain regions. The authors posited that the associated changes in microglial activity in response to acupuncture may modulate neuroinflammatory processes, and thereby reduce the reinforcing effects of ethanol. The pioneering work of Seo et al. provides a promising foundation for developing and testing animals models of acupuncture in the context of AUD.

## Conclusion

The integration of these four studies emphasized the potential of rodent PA and other adjunctive treatments to combat everyday barriers to health. The insights gained from these studies not only enhance our understanding of physiological and neural mechanisms in response to stress, high fat diet, and alcohol use, but provide further avenues for developing targeted, non-invasive therapies. As we continue to unravel the complexities of human disease, it remains essential to think outside of singular mechanisms and reductive, druggable targets. By leveraging advanced technologies like 3D-printing and exploring “alternative” therapies such as acupuncture, we can pave the way for more effective and personalized approaches to managing stress, substance use, and dietary challenges in our daily lives.
